# Systematic Review on the Safety and Tolerability of Transcranial Direct Current Stimulation in Children and Adolescents

**DOI:** 10.3390/brainsci11020212

**Published:** 2021-02-10

**Authors:** Derrick Matthew Buchanan, Thomas Bogdanowicz, Neha Khanna, Guillaume Lockman-Dufour, Philippe Robaey, Amedeo D’Angiulli

**Affiliations:** 1 Department of Neuroscience, Carleton University, Ottawa, ON K1S 5B6, Canada; nicerlab@cunet.carleton.ca (T.B.); NehaKhanna@cmail.carleton.ca (N.K.); GuillaumeLockmanDufo@cmail.carleton.ca (G.L.-D.); probaey@cheo.on.ca (P.R.); amedeo.dangiulli@carleton.ca (A.D.); 2 Neuroscience of Imagination Cognition Emotion Research Lab, Carleton University, Ottawa, ON K1S 5B6, Canada; 3 Neuropsychiatric Lab, Children’s Hospital of Eastern Ontario, Ottawa, ON K1H 8L1, Canada; 4 Department of Psychiatry, University of Ottawa, Ottawa, ON K1N 6N5, Canada

**Keywords:** tDCS, children, safety, tolerability, brain stimulation

## Abstract

Background: Transcranial direct current stimulation (tDCS) is a safe, tolerable, and acceptable technique in adults. However, there is limited evidence for its safety in youth. Although limited, there are a handful of important empirical articles that have evaluated safety and tolerability outcomes in youth. However, a synthesis of pediatric safety studies is not currently available. Objective: To synthesize objective evidence regarding the safety and tolerability of pediatric tDCS based on the current state of the literature. Methods: Our search and report used PRISMA guidelines. Our method systematically examined investigations purposefully designed to evaluate the safety, tolerability, and acceptability of tDCS in healthy and atypical youth that were submitted to three databases, from the beginning of the database to November 2019. Safety considerations were evaluated by studies utilizing neuroimaging, physiological changes, performance on tasks, and by analyzing reported and objective side effects; tolerability via rate of adverse events; and acceptability via rate of dropouts. Results: We report on 203 sham sessions, 864 active sessions up to 2 mA, and 303 active hours of stimulation in 156 children. A total of 4.4% of the active sessions were in neurotypical controls, with the other 95.6% in clinical subjects. Conclusion: In spite of the fact that the current evidence is sporadic and scarce, the presently reviewed literature provides support for the safety, tolerability, and acceptability, of tDCS in youth for 1–20 sessions of 20 min up to 2 mA. Future pediatric tDCS research is encouraged.

## 1. Introduction

Transcranial direct current stimulation (tDCS) is a non-invasive neuromodulation technique considered to be safe, tolerable, and acceptable to use in adults [1,2,3,4,5,6,7,8]. However, it is minimally researched and still controversial in pediatrics [9]. tDCS utilizes a direct, low-amplitude electrical current to enhance or depress cell membrane excitability [10]. The interest around tDCS is driven by its potential to induce neuroplastic changes and potentially aid in the treatment or management of mood disorders such as depression [11], memory deficits and motor control in Parkinson’s diseases [12], memory deterioration in Alzheimer’s diseases and dementia [13], impairments in attention-deficit/hyperactivity disorder (ADHD) [14], reduction of pain in chronic pain conditions [15], and expediting stroke recovery [16]. With the mounting experimental evidence for tDCS use in adults, interest in the application of tDCS in children is growing considerably [7,17]. Rivera-Urbina et al. [17] highlighted evidence for pediatric tDCS in epilepsy, autism, dyslexia, ADHD, and other psychiatric disorders such as depression and schizophrenia. This is further supported by the review from Lee et al. [18] which presented evidence for overall interest and efficacy of tDCS in child and adolescent psychiatric disorders. Despite the growing evidence, there is still a degree of uncertainty regarding tDCS use in children. Such reservations are primarily centered on the present lack of evidence for the short- and long-term safety and tolerability of tDCS in the developing brain.

### 1.1. Current Safety Evidence

In the last 14 years, there have been a few very important safety reviews for tDCS in adults [2,3,5,6] and children [5,7,19]. The first from Poriesz et al. [2] in 2007 reported the frequency of side effects during and after tDCS in 102 participants across 567 sessions and demonstrated that tDCS is only associated with very minor side effects such as scalp tingling (70.6%), itching (30.4%), and fatigue (35.3%). In rare cases, some minor adverse events such as headache (11.8%) or trouble sleeping emerged (0.98%). These side effects are summarized below in Table 1.

Another important review from Bikson et al. [5] extensively built on these results with nearly 7000 subjects (more than 1000 of which were exposed to 5 or more sessions), including 33,000+ total sessions: zero serious adverse events were reported. See Bikson [5] for clear criteria and a definition of serious adverse events. Bikson also reported 2800 sessions in children across nearly 500 subjects: no serious adverse events occurred. Finally, Zewdie et al. [19] reported no serious adverse events across 612 sessions in 92 child and adolescent subjects all collected from the same research centre. These children all had one or more 20 min sessions with a median amperage of 1 mA across all subjects. Similar to adults, itching (70%)/tingling (37%) were the most common side effects, followed less commonly by mild headache (25%) in perinatal stroke patients but only 9.5% in typically developing subjects.

It is clear that the evidence in children is growing. However, the review from Bikson et al. [5] also reported that less than 5% of all tDCS research was conducted with children. This is an important discrepancy in the literature that may be due to minimal safety evidence surrounding pediatric tDCS. Some authors have indirectly reported on safety and tolerability in youth, but studies specifically designed with the intent to evaluate this are scarce. Without adequate evidence for tDCS safety in youth, researchers and clinicians will remain aptly cautious to use tDCS in children. The current investigation attempted to systematically address and respond to this discrepancy.

### 1.2. The Current Study

Most safety reviews to date have focussed on the broad subjective reporting of adverse events across a wide scope of the literature. Although these articles were more inclusive, it was possible to look deeper into the safety of tDCS by reviewing literature outcomes of objective measures used to assess safety and tolerability, such as pathological changes in cognition, behaviour, or neurophysiology. These factors are of particular importance for understanding the safety of tDCS in the developing brain.

Accordingly, we conducted a systematic review of tDCS safety studies in children and adolescents. In this case, safety studies were defined as studies that assessed pathological changes in neuroimaging, physiology, and other medical evaluations such as change in performance in a given physical, cognitive, or psychological domain as primary outcomes. The aim of the present review was to investigate the current consensus on the safety and tolerability of tDCS in youth based on this scope of the literature. In line with previous reviews [2,3,5], tolerability was measured via rate and severity of side effects/adverse events (AEs), and acceptability via rate of dropouts. We hypothesized that there would be a positive consensus between the investigations such that tDCS is safe and tolerable in children and adolescents.

## 2. Materials and Methods

### 2.1. Literature Search

**Figure 1:**
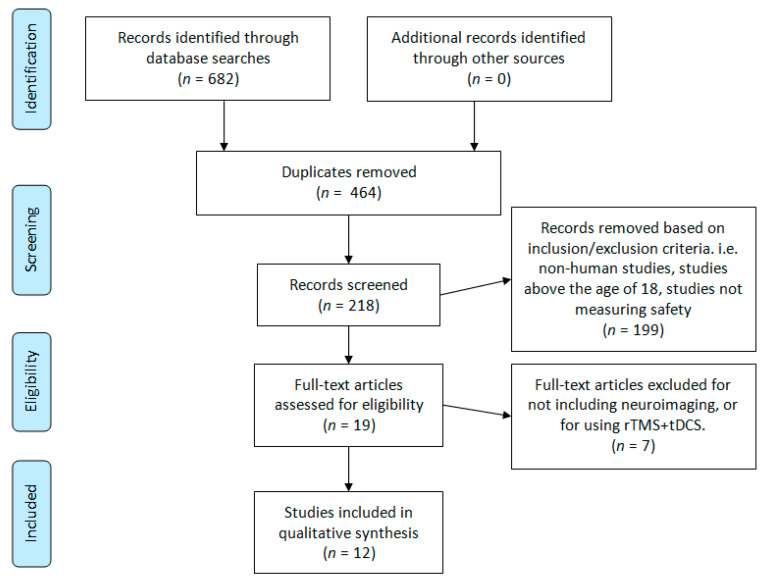
Literature search PRISMA flow diagram.

This review was designed around the following primary research question: Is tDCS objectively safe and tolerable to use in children and adolescents? To capture as many citations as possible, three scholarly databases were searched: PubMed, Scopus, and Scholar Portal. The following search terms were used for all three databases: “tDCS child safety”, “tDCS child tolerability”, “tDCS adolescent safety”, and “tDCS adolescent tolerability”. These search terms returned a total of 682 hits to be screened (Pubmed = 155, Scopus = 118, Scholar Portal = 409). A graphical depiction of the study screening and selection process is illustrated in Figure 1. Finally, references in child and adolescent tDCS review articles were investigated; this did not lead to any additional articles. The literature searches and article screening process was conducted by two authors, DMB and TB. References were screened from the origin of each database to 7 November 2019. This research design was derived in consultation with a professional university research librarian, Flavia Renon, and with due consideration of the preferred reporting in systematic review and meta-analyses (PRISMA) guidelines [20].

### 2.2. Inclusion and Exclusion Criteria

Only articles that specifically addressed the safety and tolerability of pediatric tDCS via neuroimaging plus other objective outcomes were considered. The following criteria were used: (1) English manuscripts; (2) human participants below the age of 18 (we did include two studies [21,22] where age ranged from 7 to 21 in UCP); (3) empirical research specifically reporting on child or adolescent safety, and tolerability; (4) measure and report on side effects; (5) measure and report neuroimaging outcomes. Exclusion criteria: (1) purely computational/simulated studies, (2) studies that did not directly measure safety and tolerability, and (3) studies using rTMS as a concurrent treatment (although studies using single-pulse TMS for neuronavigation/MEP measurements were included). One study was also included from Gomez et al. [23] which used tDCS and rTMS in different groups. However, we only included the data from the tDCS group. The relevant tDCS data from the study were obtained through personal communication with the corresponding author of Gomez et al. [23].

Although there are other important types of electrical brain stimulation making their way into pediatrics, such as transcranial alternating current (tACS), transcranial random noise stimulation (tRNS), and transcutaneous spinal direct current stimulation (tsDCS), the present review article was specifically focussed on articles published using tDCS ‘transcranially’ (i.e., across the cranium).

Figure 1 summarizes the results yielded from our literature search. Every abstract was initially reviewed by two authors for eligibility in our study. Our inclusion and exclusion criteria were then applied during a second abstract and partial paper review. Finally, three authors conducted a full-text review and data extraction of the remaining papers.

### 2.3. Data Collection and Outcome Measures

After reviewing the articles yielded from the database searches, a structured checklist of variables was developed: (1) metadata (i.e., authors’ names, laboratories and location, contact info, publication date and journal, one- and five-year impact factor); (2) participant demographics (age, number of males and females, atypical or neurotypical); (3) methods (randomized controlled trial, cognitive and psychological tests/questionnaires used, imaging techniques used, method of examining side effects, any other tasks performed); (4) tDCS protocol (amperage, sham vs. active, duration of stimulation, number of sessions, electrode location, electrode size, current density, content of saline, tDCS device used); (5) safety (outcome of neuroimaging, physiological changes, performance on tasks, rate and severity of side effects); (6) tolerability (derived outcome based on rate and description of AEs); (7) acceptability (derived outcome based on the rate and description of dropouts); (8) reported medications; (9) authors’ conclusions (whether pediatric tDCS is safe, tolerable, and acceptable based on their various assessments).

The main outcomes were the safety and tolerability of tDCS in youth. The secondary outcomes also included acceptability, and a verification of the influence of other variables on the primary outcomes.

### 2.4. Risk of Assessment Bias

**Figure 2:**
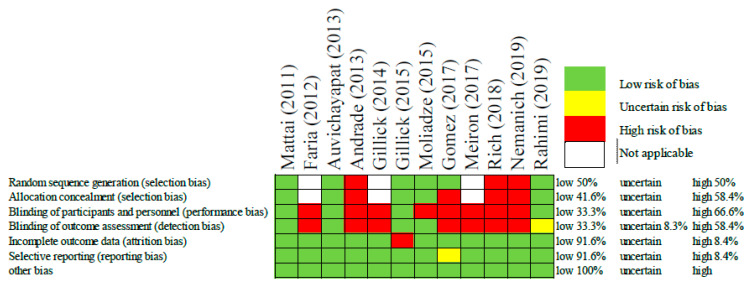
Risk of bias assessment of each included study [21,22,23,25,26,27,28,29,30,31,32,33].

In order to assess the risk of bias among the included studies, the authors used the Cochrane Collaboration’s tool for risk of bias in randomized trials [24]. This included selection bias, performance bias, detection bias, attrition bias, reporting bias, and any other bias. These biases were rated as low, uncertain, or high (Figure 2).

## 3. Results

### 3.1. Literature Search

Our initial search yielded 682 references for title and abstract review. After removing duplicates, 218 abstracts were reviewed a second time to apply our inclusion and exclusion criteria. Full-text review and data extraction were conducted for a total of 19 articles. Text reviews and data extraction were completed by DMB, TB, NK, and GLD. All text reviews and data extraction were verified by DMB. Discrepancies were resolved by AD. After applying our final inclusion and exclusion criteria, 12 papers remained for qualitative synthesis and review (Figure 1).

### 3.2. Quality of Studies

The risk of bias judgements associated with the included studies are displayed above in Figure 2. Overall, the risk of bias was high, as the majority of studies were not randomized or blinded. That being said, some of these studies had only one or two participants making randomization impractical. In this same way, other studies such as those by Faria [25] and Gillick [26], the authors were intentionally optimizing the patient’s protocol and therefore it was a necessary and a priori bias meant to improve the patient’s treatment outcomes. In these cases, the judgement of bias was considered “not applicable”. Despite the overall high risk of bias, some studies did mitigate bias through randomization, allocation concealment, and single/double blinding. All but one of the studies had low risk for attrition bias, and reporting bias. Now that these initial safety studies have been conducted, it is expected that authors will feel more comfortable employing more strict and controlled methodologies.

### 3.3. Safety and Tolerability Literature Characteristics

The results presented in Table 2 provide an overview of the available tDCS safety and tolerability research in children and adolescents. Of the 12 articles included, there were: 7 randomized controlled trials, 156 children, and 864 active tDCS sessions (0.5–2 mA) totaling 303 h of stimulation in boys and girls typically aged 6 to 17 (mean 10.75) (Table 2). It is important to remind that these numbers represent only a fraction of all the published pediatric tDCS literature [17,18,19,34], but an important fraction which specifically investigated objective measures of safety and tolerability (an extended version of Table 2, with 19 total articles, is included in the supplementary materials Table S1). In other words, there were more than 1000 sessions with rigorous safety testing. Moreover, 5 of the present studies, including a total of 69 subjects, involved repeated tDCS exposure of 10 or more sessions (max 20). This is particularly important for understanding tDCS safety as the effects of tDCS are expected to aggregate over multiple sessions and tDCS clinical treatment protocols will require several sessions [1,27,35].

One challenge in conducting the research synthesis for this article was the heterogeneity of the included research articles. For instance, several different neuropsychiatric and neurological conditions (with stroke/hemiparesis being the most common) were investigated. Some studies utilized sham while others utilized only active tDCS. Varying neuroimaging techniques were used, varying electrode locations and saline solutions were used, and side effects/adverse events were recorded with varying scales, or no scales at all, and with different repeated-measures time points. Therefore, the causal relationship between tDCS protocol and the safety/tolerability of adverse events is not always clear cut. As such, we did our best to report the results as objectively as possible.

Despite some of the heterogeneities, the overall tDCS methodology across all studies remained within the standards (i.e., 0–2 mA, 10–20 min sessions, 1–20 sessions), however Andrade et al. [30] did stimulate for 30 minutes. It is clear that some studies, such as those by the same groups of authors, built off of their previous investigations. In this way, it appears that the heterogeneities are mostly between different laboratories. Additionally, these papers included participants almost exclusively from clinical samples; only 4.4% of the subjects were neurotypical controls. Lastly, it has been demonstrated in adults that the polarizing effects of tDCS vary based on titrated amperage and the duration of the session [36,37,38]. It remains to be seen whether these varying polarizing effects are consistent in children, or whether they have an effect on safety and tolerability. Here we report on studies stimulating for 10–30 min at 0–2 mA with follow-up measures extending up to 6 months. So far, all protocols have yielded positive safety evidence.

### 3.4. Safety and Electrode Location

As part of the dosing considerations, researchers and clinicians have to be mindful of the safety and tolerability of tDCS with electrodes placed at different sites. Since each potential anatomical site may correspond to a particular physiological response, testing one site or using one montage is not entirely sufficient for drawing conclusions about each of the other potential sites. Indeed, entire literature reviews have been published on single brain regions [40]. Our review documents at least 10 unique montages corresponding to 6 brain regions: the dorsolateral prefrontal cortex (DLPFC), superior temporal gyrus (STG), Brodmann area 6 (brain region for CP5/6), Broca’s area, left medial inferior frontal gyrus (LMIFG), and the primary motor cortex (M1). That being said, in the case of Auvichayapat [29], the location varied by participant by placing the cathodal electrode over the epileptogenic region; and in Gillick [26,31], a bilateral M1 montage remained, but anode/cathode location was dependent on the lesioned hemisphere where the anode is always placed over the ipsilesional cortex. Rich [21] and Nemanich [22], on the other hand, positioned their electrodes according to the M1 TMS hotspot [41,42]. The DLPFC montage was selected to improve cognition, STG to reduce hallucinations, Brodmann area 6 to improve movement planning, LMIFG to improve language processing, and M1 to improve motor control. tDCS appears to be safe and well tolerated at each of these electrode sites. Future reviews focused on specific brain regions, such as the DLPFC review from Dedoncker [40], will make it more clear whether there are any systematic differences in safety or tolerability measures at varying electrode sites. In Section 3.8. Safety and Neuroimaging, the notion of electrode location optimization will be further discussed.

### 3.5. Electrode Size and Conducting Material

Electrode size and conducting material impact the diffusivity or locality of the stimulation via current density and therefore this information was imperative to report (Table 3). All of the present studies included precise electrode size. The majority of the reviewed studies used the common 5 × 7 (35 cm^2^) or 5 × 5 (25 cm^2^) electrodes. However, 1.09 cm^2^ HD electrode sizes were also used. Unfortunately only 58.3% articles reported the composition of their saline. Electrode preparation is important because certain solutions have been shown to be more irritable [43]. For example, water is not ideal as it contains metals that may increase the irritability of the scalp when a current is passed through them [43]. Indeed, the review from Bikson et al. [5] even went so far as to exclude studies that did not use normal saline solution because the improper preparation confounds the interpretation of tolerability and safety. Recent communications from the 2019 International Brain Stimulation Conference have actually suggested that a syringe should be used to apply saline to each electrode such that the specific amount of saline content is controlled for each subject (as opposed to soaking the entire electrode). Henceforth, studies should precisely report their electrode preparation. It is therefore recommended that authors clearly report not only electrode size, but also saline and other electrode preparation materials.

### 3.6. Safety and Medication

Assessing medication contraindications is an important criterion for safely implementing tDCS in clinical populations. There is currently a lack of medication reporting among the present studies and tDCS literature in general (Table 3). A review from McLaren (2018) clearly illustrated that medication can influence tDCS effects on tissue excitability [44] and therefore it is possible that medication may influence the safety and tolerability of tDCS. Only two of the present studies [28,33] actually reported specific medications including type and dose. Nemanich [22] provided medication type but not dose in the Supplementary Materials. In each instance, the authors admitted that it is possible that the medication influenced cortical excitability, but in any case no contraindications were reported. Other studies made a general “all or none” statement about medication use indicating either no medication, or medication as normal. It appears that the most common approach to medication involvement in tDCS protocols is to continue medication as usual. So far, this has yielded safe outcomes. However, it is possible that there remain unsafe medication contraindications that can only be elucidated with precise and consistent reporting of medication use among participants and authors. In kind with the suggestions from McLaren [44], it is advisable, where applicable, that all brain stimulation studies utilize a concomitant medication log when screening participants (a template has been provided in the Supplementary Materials; Form S1: Concomitant Medication Log).

### 3.7. Safety Indications for Specific tDCS Devices

The devices used in each study were clearly reported (five different devices used) and adverse effects do not appear to be dependent on specific tDCS devices (Table 3). That being said, it is possible that certain electrode pads, or amperage limits may lead to potentially harmful ranges in current density. In general, each manufacturer has built in common safety features to help mitigate unprecedented risk that could lead to harmful stimulation. Although the materials and features used by device manufacturers vary the physics of the method remains the same. Additionally, each device has general clearance by governing bodies such as the FDA for use as an experimental device and some for clinical purposes. As long as the devices are used properly, and the electrode materials are prepared properly, by trained individuals within the evidenced amperage range of 0–2 mA, they should each offer the same degree of tolerability. To date, there are no publications of controlled studies formally examining the tolerability of different devices.

### 3.8. Safety and Neuroimaging

All of the studies included in this review used neuroimaging to either evaluate safety or contribute to safety by mitigating risks. Several neuroimaging modalities have been used to evaluate the safety of tDCS in children including electroencephalography (EEG), magnetic resonance imaging (MRI), transcranial magnetic stimulation (TMS), event related potentials (ERP), electrocardiogram (EKG), electromyography (EMG), and electrooculography (EOG).

#### 3.8.1. EEG

A total of 67% (*n* = 8) of the studies included in our review utilized EEG to measure changes related to tDCS [23,25,28,29,30,32,33,39]. In all of these instances, no pathological changes emerged. Only 58.3% of the overall studies reported on presence or lack of epileptiform activity; no such activity emerged. Indeed, in both Faria’s [25] and Auvichayapat’s [29] epilepsy studies, epileptic discharges were actually significantly decreased (evaluated up to 4 weeks post stimulation). Moreover, Moliadze et al. [32] investigated quantitative EEG measures including spectral power analysis. They found that tDCS decreased power in all frequency bands other than alpha, and no pathological oscillations were reported. Additionally, recent studies have investigated how EEG can be used to optimize tDCS protocols in adults through current modeling [45,46]. Given the availability and low cost of EEG, it is expected that EEG combined with tDCS will become more common as an effort to optimize treatment response.

#### 3.8.2. MRI

A total of 42% (*n* = 5) of studies used MRI to evaluate/optimize safety [21,22,26,28,31]. Mattai et al. [28] indicated that they used MRI for clinical monitoring of any neurologic deterioration. They reported no changes at all. That being said, it was not clear how the authors defined neurologic deterioration. Gillick and colleagues [26,31], on the other hand, demonstrated another way that MRI could be used to assess, and enhance, the safety of tDCS through risk reduction. By deriving unique MRI head models, her team modeled tDCS current flow to maximize dose response curves and optimize stimulation intensity. This means that the children were only exposed to the necessary and hypothetically optimal amount of current of 0.7 mA. Given the potentially non-linear relationship of amperage intensity-dependent effects [37,38], the safest protocol should be optimized for dose response to avoid unnecessary exposure. This is a sound way to optimize safety in any tDCS trial. Therefore, like EEG, MRI can also serve to optimize tDCS. By collecting patient-specific MRI, the tDCS protocol can be optimized based on physical characteristics such as head and skull morphology, and pathological characteristics such as sites of brain injuries. MRI can similarly enhance TMS treatments, and be used for overall 3D neuronavigation [41].

#### 3.8.3. TMS

TMS use with MRI is also common. Rich [21] and Nemanich [22] used previously obtained MRIs from patients to create 3D head models for neuronavigation, allowing for precise locating of the motor cortex. In this way, TMS optimized tDCS safety through risk reduction by providing precise regions of interest. Additionally, motor evoked potentials (MEPs) are a clear measurement of corticospinal excitability, making it useful for monitoring pathological alterations after tDCS. MEPs were measured by 33% of the present studies. No pathological alterations were reported. However, the results from Rich et al. [21] were inconsistent and often contrary to their hypotheses. Unexpected changes in cortical excitability could be interpreted as undesirable changes in physiology and possible adverse events. However, these changes could also be interpreted as individual differences in treatment response. Nemanich [22], on the other hand, reported that 100% of participant’s in the active tDCS condition experienced a decrease in MEP, as hypothesized. Overall, optimizing tDCS stimulation intensity and location should make a safer and more suitable protocol by mitigating risks associated with individual differences.

#### 3.8.4. ERP, EKG, EMG, and EOG

ERP, EKG, EMG, and EOG were each only used once out of the 12 safety studies we reviewed. Mattai [28] provided an all or none statement, noting: “neither treatment group had significant changes in respiration, blood pressure, or heart rate during, or EEG, EKG, or MRI after tDCS” (p. 277). While encouraging, how toxicity was defined in these evaluations is not described. On the other hand, Gillick [31] clearly defined their use of EMG as an attempt to continually monitor seizures. Another advantage of this is that it allowed the various investigators to monitor seizures while remaining blinded to group assignment. No seizures or related events occurred. Lastly, Gomez [23] utilized ERP (in which EOG electrodes were simultaneously affixed) to carry out a passive oddball paradigm. Their goal was to investigate P300 amplitude and latency. ERP was only obtained from 6 subjects, each of which had normal auditory brainstem response. However, one child had no ERP response. After tDCS, P300 latency was significantly shortened, and amplitude trended toward an increase. The authors interpreted these changes as positive and in line with expectations regarding abnormal functional connectivity of the frontal lobes in ASD [23].

#### 3.8.5. Neuroimaging Conclusion

It is fair to say that the reporting of neuroimaging evidence in the context of tDCS safety is inconsistent at best. Particularly, some studies claim to have used imaging to monitor abnormal or pathological changes in physiology. However, they scarcely defined the marker or index they evaluated as an indicator of safety. On the other hand, evaluating epileptiform activity with EEG or EMG is well defined and has pathological implications that can clearly translate to tDCS safety. Future research using neuroimaging to assess the effects of tDCS should clearly define the structural or functional markers that they are measuring and what their expectations are. If these expectations are violated, it could possibly indicate a “neuroimaging adverse event”. Of course, these studies are still novel and experimental in nature and therefore defining clear clinical expectations and predictions may not be possible. In this case, the approach from Gillick [26] is highly recommended. Gillick and colleagues clearly defined the desired clinical outcomes, and outlined the expected risks, how they were mitigated, and the criteria for stopping protocol. Stopping protocol criteria is another important safety criterion that was only reported in this one study. It is recommended that future tDCS studies consider implementing and reporting the safety criteria that would result in the researcher having to stop the session (i.e., emergence of epileptiform signatures).

Outcomes related to neuroimaging and other adverse event assessments are summarized below in Table 4.

### 3.9. Safety and Medical Examination

In addition to neuroimaging multiple studies included baseline and follow-up medical examinations such as vital signs (pulse, blood pressure, temperature, respiration), blood work/biochemistry, clinical assessment of mental and functional status, and physical examination. A total of 41.6% of studies measured patients’ vital signs in relation to tDCS [21,28,29,31,33]. In general, all vital signs remained in normal age-specific range with no significant fluctuations present. However, these were again “all or none statements”; only Meiron [33] reported the details of the vitals but this was a case study in a 30-month-old child warranting in-depth details. Meiron also collected several other important data points: “The intervention was considered safe and had no negative impact on the patient’s vital signs, electrolyte levels or blood biochemistry. Neurological assessments indicated development did not change” (p. 140). Auvichayapat [29] further confirmed no clinically relevant changes in vitals from baseline to post test at 1 day, 2 days, and 1 month measurements in 36 subjects. Auvichayapat also employed a Quality of Life (QOL) questionnaire evaluating physical, social, emotional, and cognitive well-being. This was administered at the same aforementioned time points. There were no decreases in QOL. Andrade et al. [30] provided complete medical exam before and after tDCS in 14 subjects but did not provide any statement about these outcomes, though their overall study conclusions were positive and indicative of tDCS safety.

### 3.10. Safety and Neuromotor Function

One of the first neuromotor investigations in children was from Gillick et al. [31]. The aim of their study was to specifically assess safety outcomes of a single 0.7 mA tDCS session by monitoring adverse events and any potential decline in cognitive or motor function. Gillick assessed the functionality of both hands in all subjects (*n* = 11) using the Box and Blocks Test, and Grip Strength. No significant decline occurred. Moreover, from 80 1.5 mA sessions across 8 subjects with stroke/unilateral cerebral palsy (UCP), Rich et al. [21] employed the Assisting Hand Assessment (AHA) and several other secondary outcome questionnaires to investigate sensorimotor activity. Only 38% of their subjects saw improvement on the AHA. Their results for efficacy were overall inconsistent; however, there did not appear to be any deterioration of function. Also from Rich, during tDCS + bimanual training, 3 children with cerebral palsy had spasms in their more affected hand. These were considered minor adverse events and should be looked out for by future research studies involving neuromotor hand function. Nemanich [22] further evaluated a sample of 20 participants with UCP using the AHA and TMS. As noted in Section 3.8.3. TMS, active tDCS consistently decreased MEP as predicted. However, this was not correlated with increased hand function. Although there was an overall increase in AHA, none were significant between the active and sham group. In general, tDCS of the motor cortex does not appear to have any negative impact on neuromotor function in terms of side effects or deterioration of function.

### 3.11. Safety and Psychiatric/Cognitive Outcomes

Several assessment methods were employed across the included studies to monitor psychiatric outcomes such as pathological behaviour, mood changes, or cognitive outcomes such as decreased task performance. A total of 58.3% of studies [21,23,28,30,31,32,39] reported on some psychological, neurological, or neurocognitive outcomes before or after tDCS. In the case where no side effects were present, it was again common to just report an all or none statement. However, some studies did report specific cognitive side effects such as fatigue (ranging 0–31.6%), trouble concentrating (0–20%), and psychological side effects such as mood (0–42.9%) and irritability (0–35.7%). Once again, while encouraging, it would be useful for the literature if these studies provided a short description of their safety outcomes and how they defined pathological alterations in each assessment method.

A few studies employed specific questionnaires based on their samples that allowed them to evaluate systematic change in task performance or self-report. These included California Verbal Learning Test (CVLT), Wechsler Memory Scale, clinical ratings (Symptom Assessment for Positive Symptoms [SAPS]), Brief Psychiatric Rating Scale for Children, mini mental status examination (MMSE) [28]; activities of social interaction and speech, Patient Global Impression of Improvement [30]; The Token Test of Intelligence [31]; Autism Behavioural Checklist (ABC) and the Autism Treatment Evaluation Checklist (ATEC) [23]; Gap in Noise (GIN) test [39]. In general, none of the aforementioned measurements indicated any deterioration of verbal fluency, speech, memory, mental status, or behaviour. Due to the heterogeneity of these study measures they were not particularly suitable for an aggregate synthesis.

### 3.12. Reports of Adverse Events

Under the definition from Bikson [5], there were no serious adverse events in any of the 12 studies included in this review. A total of 83% of the studies included some report on adverse events and tolerability. A total of 66% of these [21,22,25,26,28,30,31,32] employed relatively congruent structured questionnaires such as the one originally proposed in the Poreisz [2] safety review. This was a positive, and it is recommended that future studies do the same. These studies investigated side effects such as itching, tingling, pain, burning, rash, blisters, fatigue, insomnia, nausea, mood, and difficulty concentrating. These questionnaires were typically self-report and included scales for severity and duration (i.e., during the session, and how long after the session). Note, the studies in Table 4 only reflect the studies that reported quantitative measures; several studies simply made qualitative statements such as “participants did not experience any adverse events”. As for the studies that reported quantitative measures, they were concurrent with adult safety reviews: the most frequent side effects in children were also tingling and itching ranging from 25 to 57.9% and 25 to 53.8%, respectively, across the included studies. This was followed by fatigue 14.3–30.7%, a burning sensation 14.3–31.6%, headache 14.3–25%, and trouble concentrating 12.5–20%. Compared to the adults from Poreisz [2] (tingling 70.6%, itching 30.4%, fatigue 35.3%, headache 11.8%, nausea 2.9%, and wakefulness 0.98%); and the children from Zewdie et al. [19] (itching (70%)/tingling (37%), mild headache (9.5–25%), burning sensation (10–20%), nausea (5%)), the results of the present review are fairly consistent. Mattai [28] and Gillick [31] were the only studies that statistically compared side effects between active and sham groups. Neither found any significant differences between groups. This is in line with the results from our group where we assessed tDCS safety and tolerability between adults and children [9]. A few other studies also clearly presented similar tolerability between active and sham groups despite not comparing them statistically. Moreover, all of these side effects generally seemed to subside with cessation of stimulation. Only rarely did they persist beyond a couple of hours. Also of note, it appears that higher stimulation intensities such as 2 mA used by Mattai [28] were associated with higher propensity for itching sensations compared to lower intensities.

The only minor adverse events that really stand out from our review are the changes in mood (42.9%) and irritability (35.7%) reported by Andrade [30]. As mood changes have not been commonly reported in tDCS, it is not clear what brought about this change in the children. Andrade addressed this discrepancy through a parent report by asking them to what degree they believed the individual side effects were related to stimulation. To that end, Andrade “noted that burning sensation, scalp pain, and redness complaints were considered by the parents as definitively related to the intervention in all cases. Headache, sleepiness, and trouble concentrating were also considered to be possibly or probably related to stimulation in 50% of the cases. Other psychological symptoms, such as acute mood change and irritability, were not reported as being related to stimulation in 66.7% and 40% of the patients, respectively” [30] (p. 1362). One notable discrepancy however is that Andrade’s study was the only one that stimulated for 30 minutes, and it also had the youngest sample of all (average age 7.57). Given the evidence for duration dependent plasticity changes [36] it is possible that 30 minutes was too long for these subjects. It is also possible that 30 minutes was too long for these subjects on account of their specific diagnoses such as PDD and AS. These populations, especially at a young age, may be more prone to mood changes and irritability than the majority of the other populations included in this review.

### 3.13. Acceptability and Rate of Dropouts

A total of 75% of the studies reported on participant attrition. In order to facilitate reviews on tDCS acceptability, and for general transparency, it is recommended that all tDCS studies make a statement about attrition. This statement should indicate “no attrition”. Or, in the event of attrition, this statement should indicate the reason, and whether it was related to stimulation. In the present review, only one subject did not complete their study protocol due to discomfort. Under this definition “rate of dropouts = acceptability”, tDCS appears to be very acceptable in children and adolescents. That being said, recent research [8,47] has demonstrated that defining acceptability by rate of dropouts can be limited. Specifically, parents of children who have underwent tDCS were interviewed regarding tDCS acceptability: for them, tDCS acceptability was primarily dependent on efficacy, availability, cost, and side effects compared to medication [8].

## 4. Discussion

In this review, we advanced the literature by providing a consensus of the leading studies evaluating tDCS safety and tolerability in children. Paradoxically, the only way to address future unknown outcomes is by conducting more tDCS research in children. The current safety results (i.e., lack of any serious adverse events, and lack of neurological/psychological damage) provide support for the continuation and extrapolation of experimental and clinical tDCS use in children. Overall, the safety evidence appears to be quite strong and consistent for 10–20 min tDCS sessions ranging from 0.5 to 2 mA in ages 5–18. Although Andrade [30] administered 30 min tDCS sessions, it was the only study to do so and therefore to be conservative the results of this review are limited to recommending 20 minutes of tDCS. Moreover, half of the investigations had subjects with a mean age <12, and half had a mean age >12. Therefore, the evidence provided in this review rather evenly supports the safety of tDCS in both children and in adolescents. All of the methodologies used in the included studies were adopted from previous adult investigations. Therefore, the positive evidence from these studies suggests that other adult tDCS protocols within the above protocol ranges could likely be safely transferred to children. That being said, studies have yet to directly compare the effects of tDCS between children and adults. Adult tDCS studies have now advanced the safety limits to up to 40 min sessions, with instances of subjects receiving more than 100 sessions [5]. Adult evidence has also extended the safety limits from 2 to 4 mA [48]. Therefore, it is expected that future pediatric tDCS research will eventually also attempt protocols up to 40 min, and more than 20 sessions. However, it will likely require very a special circumstance to see 4 mA protocols in children. It is recommended and expected that when the first of these studies occurs that rigorous safety measures will be recorded and reported.

Moreover, the safety and tolerability evidence appears to hold at least up to 4 weeks post tDCS in more than 10 repeated sessions. To that end, there still remain outstanding questions regarding potential long-term use and long-term side effects of tDCS. Thus, longitudinal studies will be of great importance to the future of the field. We encourage all future tDCS studies, regardless of primary outcome, to specifically implement components for evaluating safety and tolerability outcomes to ensure the reliability of the above results and ongoing well-being of patients. The side effect questionnaire from Poreisz [2] or Brunoni [3] should be considered a minimum standard. It seems fitting that all tDCS studies ought to evaluate side effects using the same structured questionnaire. Finally, the interpretations of these results are primarily limited to clinical populations, as only 4.4% of included subjects were neurotypical. This suggests a further need for investigation into healthy populations. For children who are being treated clinically, it has been demonstrated that the benefit largely outweighs the risks. However, the same might not be so easy to say for healthy children. Based on the current evidence, it does not appear that healthy children experience tDCS in a different manner to children in the clinical groups; however, the sample sizes are still too small to draw any strong conclusions.

### 4.1. Pediatric tDCS Safety Timeline

tDCS really started gaining traction in adults in the late 1990s and early 2000s. It took approximately one decade for this knowledge to successfully transfer to adolescents and children. In 2011 Mattai et al. [28] published what appears to be the first clinical application of tDCS in youth; and rightfully so, it had an emphasis on safety and tolerability. Now, 10 years after Mattai’s initial publication, the potential for tDCS in pediatric neurology and neuropsychiatry is starting to be realized and accepted [8]. From 2011 to 2021, several hundred papers have been published about tDCS in children. However, only a limited number evaluated and reported safety outcomes, such as those reviewed in the present article. Mattai actually had a relatively small sample size of 12, but their mostly adolescent subjects were exposed to a total of 125 active tDCS sessions. Two years later, Auvichayapat et al. [29] published an epilepsy study (*n* = 36) but each subject only received a single session of tDCS. Also in 2013, Andrade et al. [30] conducted a study with 140 active sessions in 14 children with neurodevelopmental issues. Notably, Andrade’s sample has the youngest mean age (7.57) of all the studies we reviewed (not including the infant case study). This was less than half the average age of Mattai’s sample from two years previous (16.37). Andrade’s sample was also the only one that underwent 30 minutes tDCS sessions. Sample size and number of sessions in these safety trials continued to grow until they peaked with Gomez [23] in 2017, with 15 subjects receiving a total of 300 active sessions.

The knowledge progression seen from Gillick 2014 to the present also significantly advanced evidence of the safety and tolerability of pediatric tDCS. Gillick and her lab are responsible for 1/3 of the included studies. The studies began in 2014 [26], with only a single subject and a single 10 min session; and then in 2015 [31], with 11 subjects also receiving only a single 10 min session. Then in 2018 and 2019, Gillick was senior author with Rich [21] and Nemanich [22] as leads, respectively. From Rich in 2018, we saw an increase in duration of sessions from 10 to 20 min, and number of sessions from 1 to 10 for 8 subjects. Then in 2019, Nemanich provided an even larger sample of 20 subjects each receiving 10 sessions of 20 min each. Nemanich’s trial also included the largest sample of 100 sham tDCS sessions, to date. These four studies had perhaps the most consistent reporting of side effects and adverse events considering they were all published under the seniority of Gillick.

At the same time as Gillick, Kirton’s laboratory also provided significant advancements in pediatric tDCS in neurotypical controls [49,50] as well as stroke [51]. The contributions from Kirton were all rigorously controlled and saw a similar scientific progression as Gillick such as increased number of sessions and additional control arms. Also similar to Gillick, all of the papers from Kirton’s supervision maintained rather consistent reporting styles making them easier to synthesize and interpret. The methodology of Kirton’s papers also included routine tolerability side effect questionnaires, often TMS motor mapping, and occasionally other assessments like vital signs and cognitive evaluations. That being said, the reported results from Kirton [49,50,51] tended to focus on the efficacy of tDCS and less on changes in objective safety measures like neuroimaging (some of these extended results are available in Table S1).

In the 10 years spanning all of the reviewed trials, the expected and experienced side effects have consistently remained only mild sensations of itching, tingling, burning, or pain. That being said, the distinction between sensations such as itching, tingling, burning, or pain on the scalp are not well defined and therefore responses may be biased by the frame of the question. This is why many of the side effect questionnaires employed today ask specifically about each individual sensation. On that note, it was surprising that other potential sensory side effects related to vision or hearing were scarcely investigated. In addition to using a consistent side effect questionnaire, future researchers should also consider defining their desired clinical outcomes and clear criteria for stopping protocol. Studies using neuroimaging as a marker of safety in terms of detecting abnormal function after tDCS should clearly define what they consider abnormal (i.e., the type of signature, the number of standard deviations required to be abnormal). The same should be indicated for cognitive, psychiatric, motor, and other outcomes specific to the authors’ hypotheses. This would really improve the level of interpretability and aggregation of the safety results across the literature.

### 4.2. The Safety of Remote and At-Home tDCS

Recent trends in tDCS research have focused on at-home use. Indeed, multiple studies have found good compliance and outcomes using an at-home tDCS approach [52,53,54]. Although this raises some obvious safety concerns, Charvet et al. [55] and Knotkova et al. [56] have published practical guidelines for at home or remotely supervised tDCS use. These are admirable steps forward and will help researchers and clinicians safely implement tDCS at home. This is particularly important in the current climate of COVID-19. A recent guideline was published by Bikson et al. [57] to outline the necessity and importance of safely implementing and continuing tDCS and TMS treatments during the pandemic. That being said, the biggest risks associated with tDCS are not likely to occur in clinical trials or clinical applications, but rather among consumers pursuing self-prescribed at-home tDCS. If used improperly, tDCS may cause burns and potentially other unforeseen adverse events.

At the moment, consumer tDCS devices are readily available for less than $200 via ecommerce websites such as Amazon; and consumer reviews seem to indicate that it is in demand. A quick YouTube search of at-home tDCS will reveal videos with tens of thousands of views further attesting to consumer interest and demand. Even parents of children with ADHD have expressed their desire to pursue tDCS treatment [8]. Despite expert panels [58], these devices are available for sale without any governing authority or regulation. Aside from the warranted safety concerns, this poses a serious healthcare dilemma. At the moment, tDCS is not a readily available treatment option in most hospitals or outpatient treatment centres, but it is available for unregulated consumer purchase online. This means that individuals who desire tDCS treatment will primarily have to pursue it on their own without the guidance of a medical professional. It would seem that the adoption of tDCS into community medicine would help to ensure the safety of those pursuing tDCS as a treatment option. Therefore, there is a consistent onus on researchers to disseminate evidence into community medicine and to educate clinicians on how to properly implement tDCS into their practice. It is evident that when used properly tDCS has the capability of being a safe and effective medical intervention; there is no reason why patients should have to put themselves at risk by pursuing this treatment on their own through consumer devices.

### 4.3. Dose Optimization

Another recent trend in tDCS research is dosage, such as individualized amperage and electrode montage based on MRI, EEG, or TMS [11,36,37,46,59,60,61,62,63]. This research has revealed that tDCS, much like medication, dosage and titration can be optimized based on individual characteristics such as age and weight, but also skull and brain morphology. Arguably, individualized tDCS protocols should radically improve treatment efficacy and subsequently safety. If individualized, tDCS is considered the highest level of safety precaution, then consumer-prescribed tDCS would be the least. In the present review, five studies used methods to individualize tDCS, such as Auvichayapat [29] placing the electrode over the epileptogenic focus, or Rich [21] using the TMS motor hot spot and MEP threshold. Although the other seven studies did not individualize their tDCS protocols, they collectively provided sound evidence for the general safety of tDCS at multiple relevant brain regions such as the DLPFC, STG, and M1. Overall, similar to adults, it is clear that the optimal stimulation target site for tDCS is totally dependent on the desired clinical outcomes and the subject specific characteristics. That said, a few things can definitely be agreed upon: in stroke or neuromotor studies, and epilepsy studies, individualization of stimulation parameters, especially location, is the gold standard. This is true in adults and it holds true in youth.

### 4.4. Limitations of the Literature and This Review

One limitation of this review that should be noted is that the researchers involved in the literature review were not blinded. It is possible that this could have led to some selection bias. However, we are confident that our method was robust and in line with our inclusion and exclusion criteria. The interpretation of the present results also needs to bear in mind the potential bias as reported in Section 2.4. Risk of Bias Assessment. Given the novelty of pediatric tDCS, many of the earlier studies were designed without blinding, placebos, or randomization. This was mostly performed for safety, ethicality, and likely recruitment reasons, but it is possible that these biases may have affected the results of those individual studies and therefore left traces of bias in review papers such as this. That being said, it seems likely that such biases would bear more of an impact on the interpretation of efficacy; here we are interested specifically in objective measures of safety, and in this regard all of the authors made similar positive conclusions. Indeed, in the discussion and conclusions of the included papers, each of the authors generally make reference to progressing to larger sample sizes. Evidence of this is clear from Gillick’s lead in four articles discussed in Section 4.1. Pediatric tDCS Safety Timeline. Having completed their initial safety investigations this is the logical scientific progression.

Finally, interpreting tDCS tolerability is further complicated by anode and cathode locations. If an adverse event occurred, it is not immediately clear whether it was caused by one or both of the electrodes. In the case of a physical side effect such as burning, blistering, and itching, it is however clear, and authors should consider reporting at which electrode site the side effects occurred; Moliadze et al. [32] were the only authors to do this. For this reason, it was not possible to report on systematic differences related to anode or cathode safety/tolerability.

## 5. Conclusions

Overall, we report on 203 sham sessions, 864 active sessions up to 2 mA, and 303 active hours of stimulation in 156 participants. A total of 4.4% of the active sessions were in neurotypical controls, with the other 95.6% in clinical subjects. Moreover, it is evident by the participation of nearly 100 authors from 10 countries across 4 continents that the safety of pediatric tDCS is of global interest. Internationally, tDCS appears to be safe and highly tolerable, with only minor levels of discomfort in a minority of children and no serious adverse events. tDCS also appears to be very acceptable, with nearly all enrolled subjects completing their respective protocol. Therefore, based on the present update, tDCS appears ready for widespread research and increased clinical use in pediatric neurology and neuropsychiatry.

## Figures and Tables

**Table 1:** Table of common, uncommon, and rare side effects previously investigated in tDCS for adults and children.

Common	Uncommon	Rare and/or Serious
Tingling	Headache	Visual perceptual changes
Itching	Nausea	Transient decrease in attention
Redness at site	Small blisters at the site	Transient decrease in memory
Discomfort	Nervousness	Difficulty concentrating
Mild burning sensation	Feeling sleepy or wakeful	Mood changes

**Table 2:** tDCS exposure for age and population.

Study	*n* (Total)	*n* (Active)	*n* (Sham)	*n* (Min)	Amperage (mA)	Age (Years)	Population	RCT
Mattai (2011) [28]	12 175	5 (7) 125	3 (2) 50	20 2500	2, sham	10–17 (16.37)	COS	Yes
Faria (2012) [25]	2 6	2 4	2 2	15 60	0.5, 1, sham	7–11 (9)	CSWS/LKS	No
Auvichayapat (2013) [29]	36 36	20 (7) 27	6 (3) 9	20 540	1	6–15 (11.46)	Epilepsy	Yes
Andrade (2013) [30]	14 140	10 (4) 140	0	30 4200	2	7–12 (7.57)	ELD/PDD-NOS/AS/GD	No
Gillick (2014) [26]	1 1	1 1	0	10 10	0.7	10	Stroke/Hemiparetic CP	Yes
Gillick (2015) [31]	11 11	3 (2) 5	1 (5) 6	10 50	0.7	7–18 (14)	Congenital hemiparesis	Yes
Moliadze [32] (2015)	19 57	8 (11) 38	8 (11) 19	10 380	1	11–16 (13.9)	Neurotypical	Yes
Gomez (2017) [23]	15 300	10 (5) 300	0	20 6000	1	5–10 (7.7)	ASD	Yes
Meiron (2017) [33]	1 10	1 10	0	20 200	0.1–1	2.5	Epileptic encephalopathy	No
Rich (2018) [21]	8 80	3 (5) 80	0	20 1600	1.5	7–21 (13.4)	Perinatal stroke/UCP	No
Nemanich (2019) [22]	20 200	5 (5) 100	4 (6) 100	20 2000	0.7, sham	7–21 (12.75)	UCP via hemispheric stroke/PVL	Yes
Rahimi (2019) [36]	17 51	9 (8) 34	9 (8) 17	20 680	1, sham	9–12 (10.35)	Dyslexia	No
Overall	156 1067	77 (54) 864	33 (35) 203	18,220 303 (h)	0.5, 0.7, 1, 1.5, 2, sham	6–17 (10.75)		

Table 2 summarizes the extent of tDCS exposure for age and population. *n* (total) is the total number of individual participants on top and the total quantity of tDCS sessions below. *n* (active) and *n* (sham) represent the number of males and females (in parentheses) who received active versus sham tDCS and the number below indicates the quantity of tDCS sessions. *n* (min) indicates the duration of each single tDCS session on top, with the number below representing the number of total minutes of *active* tDCS exposure in each study. Amperage indicates the level of current used in the given sessions. Age is identified first as a range on top and below as an average. Population includes children and adolescents aged 6–18 years (COS = Childhood Onset Schizophrenia; CSWS = Continuous Spikes and Waves during Sleep—Rare Epilepsy; LKS = Landau–Kleffner Syndrome; ELD = Expressive Language Disorder; PDD-NOS = Pervasive Developmental Disorder Not Otherwise Specified; AS = Asperger Syndrome; GP = Global Dyspraxia; CP = cerebral palsy; UCP = unilateral cerebral palsy; PVL = periventricular leukomalacia). RCT indicates whether the experiment was a randomized controlled trial.

**Table 3:** Author demographic and materials used.

Study	Location of Research and Device Used	Medications Reported	Electrode Location	Electrode Size (cm^2^)	Sponges Soaked With
Mattai [28] (2011)	USA Phoresor II 850	Medication as usual. The atypical antipsychotic clozapine ranging from 100 to 450 mg. Antipsychotic medication may have altered cortical excitability in the cohort, thereby preventing the functional effects of tDCS.	Bilateral anodal DLPFC to improve cognitive difficulty or bilateral cathode STG to reduce hallucinations.	25	Tap water or normal saline
Faria [25] (2012)	Portugal/USA Phoresor 850	None to be reported.	Cathode placed at CP6/CP5.	1.09	Skin Pure/Electrogel
Auvichayapat [29] (2013)	Thailand/USA Soterix constant current	Medication as usual. Antiepileptic medication regimens.	The cathodal electrode was placed over the epileptogenic focus. The anodal electrode was placed over the contralateral shoulder area.	35	Normal saline
Andrade [30] (2013)	Brazil/USA Striat	None to be reported.	Anode was positioned in the Broca area (mid-left inferior frontal gyrus) and the cathode in the right supraorbital area. In patient 3, the electrodes were placed in the opposite hemisphere (however, in the same location).	35	Normal saline
Gillick [26] (2014)	USA/Canada Soterix LTE	The subject was developmentally normal, did not have epilepsy, and was not taking any neuroactive medications.	Cathode placed over the contralesional motor cortex and anode over the ipsilesional supraorbital region with the intent to inhibit contralesional effects upon the ipsilesional cortex.	35	Normal saline
Gillick [31] (2015)	USA Soterix LTE	One participant was taking Levetiracetam.	Primary motor cortex. The cathode rubber electrode was placed over the M1 FDI hotspot of the non-lesioned hemisphere, and the anode rubber electrode was placed over the M1 FDI hotspot of the lesioned hemisphere.	35	Normal saline/disinfected
Moliadze [32] (2015)	Germany/Russia neuroConn constant current	None to be reported. None of the subjects took any psychoactive drugs, smoked, or drank alcohol regularly.	Anodal, cathodal, and sham tDCS were applied over the left primary motor cortex (M1, over C3). The reference electrode was placed over the contralateral orbit.	35	Normal saline/70% cellulose 30% cotton sponge
Gomez [23] (2017)	Cuba Neuroconn tDCS stimulator	Medication as usual. Only patients with no changes in their therapeutic scheme, pharmacologically or non-pharmacologically, were accepted. Patients who needed any change were excluded from the trial.	Cathode was placed over area F3 (based on the 10/20 international EEG electrode system), with the anode over the proximal right arm.	Not specified	0.9% NaCl solution
Meiron [33] (2017)	Israel Soterix LTE	The infant was given anticonvulsants (Phenytoin and Phenobarbital) with no effect. Epileptic medication then changed to Clonazepam (1.5 mg/day), Vigabatrin (500 mg/day) and Topiramate (100 mg/day).	A 4 × 1 ring configuration was applied so that the cathode was placed over the right temporal area and the four anodes were situated around the cathode. The right hemisphere received 90% of current.	1.09	Not specified
Rich [21] (2018)	USA Soterix LTE	No patients reported use of medications acting on the central nervous system for seizure control. No patients had phenol or botulinum toxin injections in the six months prior to the experiment.	The cathode was placed on the TMS-derived hotspot (primary motor cortex) of the non-lesioned hemisphere. The reference electrode was placed on the contralateral supraorbital region.	25	Not specified
Nemanich [22] (2019)	USA Soterix LTE	None to be reported.	The cathode was placed over the TMS-derived motor hotspot of the contralesional hemisphere, and the anode was placed over the contralateral forehead.	Not specified	Not specified
Rahimi [36] (2019)	Iran Electrical Brain Stimulator Neurostim	None to be reported.	Anode on the left superior temporal gyrus (STG), with the cathode either placed on right STG or on the right shoulder.	25	0.9% NaCl solution

Table 3 summarizes the author demographics, the tDCS materials that were used, any potential medications reported, and the anode/cathode electrode placement. Location of research and device used indicate the country or countries where the research was conducted and the brand/model of tDCS device that was used. Medications reported summarizes any psychoactive substances that subjects may have been exposed to during the trial. Electrode location provides a description of the placement of the anode and cathode electrodes. Electrode size indicates the surface area of the electrode in centimeters squared. Sponges soaked with indicates the materials used to prepare the electrode sponges.

**Table 4:** Adverse events reports.

Study	Neuroimaging AEs	Clinical AEs	Psychiatric AEs	Cognitive AEs	Physical AEs	Dropouts
Mattai (2011) [28]	0%	0%	0%	Fatigue 30.7%	Itching 53.8% Tingling 46.1%	0%
Faria (2012) [25]	0%	-	-	-	Felt something 40%	0%
Auvichayapat (2013) [29]	0%	0%	-	-	Skin rash 2.7%	0%
Andrade (2013) [30]	0%	0%	Mood 42.9% Irritability 35.7%	Fatigue 14.3% Trouble Concentrating 14.3%	Tingling/Itching 28.6% Burning 14.3% Headache 14.3% Scalp pain 7.1%	0%
Gillick (2014) [26]	-	-	-	-	0%	0%
Gillick (2015) [31]	0%	0%	0%	Fatigue 20% Trouble Concentrating 20%	Itching and tingling 0% Burning 16.7%	8.3%
Moliadze [32] (2015)	0%	-	5.3%	Fatigue (A) 15.8% Fatigue (C) 31.6	Tingling (A) 31.6% Burning (A) 31.6% Pain (mild) (A) 15.5% Tingling (C) 57.9% Burning (C) 26.3% Pain (mild) (C) 10.5%	-
Gomez (2017) [23]	0%	0%	0%	0%	-	-
Meiron (2017) [33]	0%	0%	-	-	0%	0%
Rich (2018) [21]	0%	0%	0%	Fatigue 25% Trouble Concentrating 12.5%	Felt something 37.5% Itchiness 25% Tingling 25% Headache 25%	0%
Nemanich (2019) [22]	0%	0%	-	-	0%	-
Rahimi (2019) [36]	0%	-	-	0%	-	0%

Table 4 summarizes adverse events reported by the included studies based on several domains. Neuroimaging = any pathological changes; Clinical = any decline in prognosis, any abnormalities from medical exams; Psychiatric = any changes in behaviour, mood, or mental status; Cognitive = any decline in cognitive function; Physical = any physical side effects, Dropouts = any participant attrition due to stimulation. The table only includes minor adverse events that did occur and were reported. If a study reported a specific value, it was included in the table. If the study did not report the value, a “-” was used. If the outcome was measured but there was nothing to report, 0% was used. In the article by Moliadze [32] the authors reported side effects separately based on (A) anode and (C) cathode locations.

## Data Availability

The database is submitted for publication and therefore we will send it only privately upon request until it is made public and published for everyone to access.

## Supplementary Materials

The following are available online at https://www.mdpi.com/2076-3425/11/2/212/s1, Table S1: Extended table for tDCS exposure for age and population, Form S1: Concomitant Medication Log.
