# Supplementary material for: Systematic Review on the Safety and Tolerability of Transcranial Direct Current Stimulation in Children and Adolescents
Source: Brain Sci. 2021 Feb 10;11(2):212. doi: 10.3390/brainsci11020212 (PMC7916366; doi:10.3390/brainsci11020212)
Supplement: Supplementary file 1 [file brainsci-11-00212-s001.pdf]

<u>Supplementary data</u>

Table S1. Extended table for tDCS exposure for age and population

Study	*n*<br>(total)	*n*<br>(active)	*n*<br>(sham)	*n*<br>(min)	Amperage<br>(mA)	Age<br>(years)	Population	RCT
Mattai<br>(2011) [1]	12<br>175	5(7)<br>125	3(2)<br>50	20<br>2500	2, sham	10-17<br>(16.37)	COS	Yes
Faria<br>(2012) [2]	2<br>6	2<br>4	2<br>2	15<br>60	.5, 1, sham	7-11<br>(9)	CSWS/LKS	No
Auvichayapat<br>(2013) [3]	36<br>36	20(7)<br>27	6(3)<br>9	20<br>540	1	6-15<br>(11.46)	Epilepsy	Yes
Andrade<br>(2013) [4]	14<br>140	10(4)<br>140	0	30<br>4200	2	7-12<br>(7.57)	ELD/PDD-<br>NOS/AS/GD	No
Gillick<br>(2014) [5]	1<br>1	1<br>1	0	10<br>10	0.7	10	Stroke/Hemi-<br>paretic CP	Yes
Gillick<br>(2015) [6]	11<br>11	3(2)<br>5	1(5)<br>6	10<br>50	0.7	7-18<br>(14)	Congenital<br>Hemiparesis	Yes
Moliadze<br>(2015) [7]	19<br>57	8(11)<br>38	8(11)<br>19	10<br>380	1	11-16<br>(13.9)	Neurotypical	Yes
Ciechanski<br>(2016) [8]	24<br>72	11(7)<br>54	3(3)<br>18	20<br>1080	1,2, sham	13-15<br>(14)	Neurotypical	Yes
Kirton<br>(2017) [9]	23<br>230	8(4)<br>120	7(4)<br>110	20<br>2400	1, sham	6-18<br>(11.8)	Unilateral<br>perinatal<br>stroke	Yes
Gómez<br>(2017) [10]	15<br>300	10(5)<br>300	0	20<br>6000	1	5-10<br>(7.7)	ASD	Yes

Meiron (2017) [11]	1<br>10	1<br>10	0	20<br>200	0.1-1	2.5	Epileptic Encephalopathy	No
Gillick (2018) [12]	20<br>200	5(5)<br>100	4(6)<br>100	20<br>2000	0.7, sham	7-21<br>(12.75)	UCP via Hemispheric stroke/PVL	Yes
Rich (2018) [13]	8<br>80	3(5)<br>80	0	20<br>1600	1.5	7-21<br>(13.4)	Perinatal stroke/UCP	No
Costanzo (2018) [14]	11<br>198	1(10)<br>198	0	20<br>3960	1	10-15<br>(13.9)	Anorexia Nervosa	No
Cole (2018) [15]	24<br>120	9(7)<br>80	2(6)<br>40	20<br>1600	1, sham	12-18<br>(15.5)	Neurotypical	Yes
Costanzo (2019) [16]	26<br>468	5(8)<br>234	6(7)<br>234	20<br>4680	1, sham	10-17<br>(13.75)	Dyslexia	Yes
Nemanich (2019) [17]	Same sample as Gillick (2018) [12]				0.7, sham	7-21<br>(12.75)	UCP via Hemispheric stroke/PVL	Yes
Ciechanski (2019) [18]	1<br>10	0(1)<br>10	0	20<br>200	1.5	17	Stroke Hemiparesis	No
Rahimi (2019) [19]	17<br>51	9(8)<br>34	9(8)<br>17	20<br>680	1, sham	9-12<br>(10.35)	Dyslexia	No
Overall	265<br>2165	111(91)<br>1560	51(55)<br>605	32140<br>535 (h)	0.5, 0.7, 1, 1.5, 2, sham	2.5-21<br>(12)		

This table summarizes the extent of tDCS exposure for age and population. *n* (total) is the total number of individual participants on top and the total quantity of tDCS sessions below. *n* (active) and *n* (sham) represents the number of males and females (in

parentheses) who received active versus sham tDCS and the number below indicates the quantity of tDCS sessions. *n* (min) indicates the duration of each single tDCS session on top, with the number below representing the number of total minutes of *active* tDCS exposure in each study. Amperage indicates the level of current used in the given sessions. Age is identified first as a range on top and below as an average. Population includes children and adolescents aged 6–18 years (COS = Childhood Onset Schizophrenia; CSWS = Continuous Spikes and Waves during Sleep–Rare Epilepsy; LKS = Landau-Kleffner Syndrome; ELD = Expressive Language Disorder; PDD-NOS = Pervasive Developmental Disorder Not Otherwise Specified; AS = Asperger Syndrome; GP = Global Dyspraxia; CP = Cerebral Palsy; UCP = Unilateral Cerebral Palsy; PVL = Periventricular leukomalacia). RCT indicates if the experiment was a randomized controlled trial.

Table S1 References

1. Mattai, A.; Miller, R.; Weisinger, B.; Greenstein, D.; Bakalar, J.; Tossell, J.; David, C.; Wassermann, E.M.; Rapoport, J.; Gogtay, N. Tolerability of transcranial direct current stimulation in childhood-onset schizophrenia. *Brain Stimul.* **2011**, *4*, 275–280, doi:10.1016/j.brs.2011.01.001.

2. Faria, P.; Fregni, F.; Sebastião, F.; Dias, A.I.; Leal, A. Feasibility of focal transcranial DC polarization with simultaneous EEG recording: Preliminary assessment in healthy subjects and human epilepsy. *Epilepsy Behav.* **2012**, *25*, 417–425, doi:10.1016/j.yebeh.2012.06.027.

3. Auvichayapat, N.; Rotenberg, A.; Gersner, R.; Ngodklang, S.; Tiamkao, S.; Tassaneeyakul, W.; Auvichayapat, P. Transcranial

direct current stimulation for treatment of refractory childhood focal epilepsy. *Brain Stimul.* **2013**, *6*, 696–700, doi:10.1016/j.brs.2013.01.009.

4. Andrade, A.C.; Magnavita, G.M.; Allegro, J.V.B.N.; Neto, C.E.B.P.; Lucena, R. de C.S.; Fregni, F. Feasibility of Transcranial Direct Current Stimulation Use in Children Aged 5 to 12 Years. *J. Child Neurol.* **2013**, *29*, 1369-1365, doi:10.1177/0883073813503710.

5. Gillick, B.T.; Feyma, T.; Menk, J.; Krach, L.E. Safety of transcranial direct current stimulation in pediatric hemiparesis: determination of the method for locating the optimal stimulation site. *Brain Stimul.* **2014**, *7*, e12, doi:10.1016/j.brs.2014.01.042.

6. Gillick, B.T.; Feyma, T.; Menk, J.; Usset, M.; Vaith, A.; Wood, T.J.; Worthington, R.; Krach, L.E. Safety and feasibility of transcranial direct current stimulation in pediatric hemiparesis: randomized controlled preliminary study. *Phys. Ther.* **2015**, *95*, 337–349, doi:10.2522/ptj.20130565.

7. Moliadze, V.; Andreas, S.; Lyzhko, E.; Schmanke, T.; Gurashvili, T.; Freitag, C.M.; Siniatchkin, M. Ten minutes of 1mA transcranial direct current stimulation was well tolerated by children and adolescents: Self-reports and resting state EEG analysis. *Brain Res. Bull.* **2015**, *119*, 25–33, doi:10.1016/j.brainresbull.2015.09.011.

8. Ciechanski, P.; Kirton, A. Transcranial Direct-Current Stimulation Can Enhance Motor Learning in Children. *Cereb. Cortex* **2016**, *43*, bhw114, doi:10.1093/cercor/bhw114.

9. Kirton, A.; Ciechanski, P.; Zewdie, E.; Andersen, J.; Nettel-Aguirre, A.; Carlson, H.; Carsolio, L.; Herrero, M.; Quigley, J.; Mineyko, A.; et al. Transcranial direct current stimulation for children with perinatal stroke and hemiparesis. *Neurology* **2017**, *88*, 259–267, doi:10.1212/WNL.0000000000003518.

10. Gómez, L.; Vidal, B.; Maragoto, C.; Morales, L.M.; Berrillo, S.; Cuesta, H.V.; Baez, M.; Denis, M.; Marín, T.; Cabrera, Y.; et al. Non-invasive brain stimulation for children with autism spectrum disorders: A short-term outcome study. *Behav. Sci. (Basel).* **2017**, *7*, 63, doi:10.3390/bs7030063.

11. Meiron, O.; Gale, R.; Namestnic, J.; Bennet-Back, O.; David, J.; Gebodh, N.; Adair, D.; Esmaeilpour, Z.; Bikson, M. High-Definition transcranial direct current stimulation in early onset epileptic encephalopathy: a case study. *Brain Inj.* **2018**, *32*, 135–143, doi:10.1080/02699052.2017.1390254.

12. Gillick, B.; Rich, T.; Nemanich, S.; Chen, C.Y.; Menk, J.; Mueller, B.; Chen, M.; Ward, M.; Meekins, G.; Feyma, T.; et al.

Transcranial direct current stimulation and constraint-induced therapy in cerebral palsy: A randomized, blinded, sham-controlled clinical trial. *Eur. J. Paediatr. Neurol.* **2018**, *22*, 358–368, doi:10.1016/j.ejpn.2018.02.001.

13. Rich, T.L.; Nemanich, S.; Chen, M.; Friel, K.; Feyma, T.; Krach, L.; Nawshin, T.; Meekins, G.; Gillick, B.T. Transcranial direct current stimulation (tDCS) paired with occupation-centered bimanual training in children with unilateral cerebral palsy: A preliminary study. *Neural Plast.* **2018**, *2018*, 1-14, doi:10.1155/2018/9610812.

14. Costanzo, F.; Menghini, D.; Maritato, A.; Castiglioni, M.C.; Mereu, A.; Varuzza, C.; Zanna, V.; Vicari, S. New Treatment Perspectives in Adolescents With Anorexia Nervosa: The Efficacy of Non-invasive Brain-Directed Treatment. *Front. Behav. Neurosci.* **2018**, *12*, doi:10.3389/fnbeh.2018.00133.

15. Cole, L.; Giuffre, A.; Ciechanski, P.; Carlson, H.L.; Zewdie, E.; Kuo, H.-C.; Kirton, A. Effects of High-Definition and Conventional Transcranial Direct-Current Stimulation on Motor Learning in Children. *Front. Neurosci.* **2018**, *12*, doi:10.3389/fnins.2018.00787.

16. Costanzo, F.; Rossi, S.; Varuzza, C.; Varvara, P.; Vicari, S.; Menghini, D. Long-lasting improvement following tDCS treatment combined with a training for reading in children and adolescents with dyslexia. *Neuropsychologia* **2019**, *130*, 38–43, doi:10.1016/j.neuropsychologia.2018.03.016.

17. Nemanich, S.T.; Rich, T.L.; Chen, C.-Y.; Menk, J.; Rudser, K.; Chen, M.; Meekins, G.; Gillick, B.T. Influence of Combined Transcranial Direct Current Stimulation and Motor Training on Corticospinal Excitability in Children With Unilateral Cerebral Palsy. *Front. Hum. Neurosci.* **2019**, *13*, doi:10.3389/fnhum.2019.00137.

18. Ciechanski, P.; Carlson, H.L.; Herrero, M.; Lane, C.; MacMaster, F.P.; Kirton, A. A Case of Transcranial Direct-Current Stimulation for Childhood Stroke Hemiparesis: A Brief Report. *Dev. Neurorehabil.* **2019**, *23*, 133-136. doi:10.1080/17518423.2019.1655678.

19. Rahimi, V.; Mohamadkhani, G.; Alaghband-Rad, J.; Kermani, F.R.; Nikfarjad, H.; Marofizade, S. Modulation of temporal resolution and speech long-latency auditory-evoked potentials by transcranial direct current stimulation in children and adolescents with dyslexia. *Exp. Brain Res.* **2019**, *237*, 873–882, doi:10.1007/s00221-019-05471-9.

Form S1.

Concomitant Medication Log

CONCOMITANT MEDICATIONS SOURCE DOCUMENT	
**REB#:**	**Participant ID#:** ____________________
**Study Name: Transcranial Direct Current Stimulation: A safety study in children and adolescents**	

Dose Frequency **Route** **Dose Units**

BID = twice daily<br>TID = 3 times/day<br>QID = 4 times/day<br>q2h = every 2 hours<br>q4h = every 4 hours	Q6h = every 6 hours<br>Q8h = every 8 hours<br>Qam = one dose in morning<br>Qpm = one dose in evening	QD = once daily<br>HS = at bedtime<br>PRN = as needed<br>OTH = other<br>UNK = unknown	IM – intramuscular<br>IN – intranasal<br>INH – inhaled<br>IT – intrathecally<br>IV - intravenous	PO – oral<br>SC – subcutaneous<br>TOP – topical<br>OTIC – by ear<br>OTH – other, specify	g = gram<br>gtt = drop<br>mcg = microgram<br>mcL = microliter	mg = milligram<br>mL = milliliter<br>oz = ounce<br>SPY = spray<br>supp = suppository	TBSP = tablespoon<br>tsp = teaspoon<br>OTH = other, specify<br>UNK = unknown

COMPLETE AT INITIATION OF THERAPY									COMPLETE AT DISCONT THERAPY / END OI		
MEDICATION / NON-	INDICATION (If given for an	BASELINE MED?	DOSE (indicate	FREQUENCY	ROUTE	START DATE	REPORTER INITIALS	REPORTER DATE	STOP DATE	CONTINUING AT END OF	REPO INIT

**MEDICATION THERAPY**	AE, enter the exact term detailed on the AE form)	1 – Yes 2 – No	units)			*(yyyy-mmm-dd)*		*(yyyy-mmm-dd*	*(yyyy-mmm-dd)*	**STUDY?** 1 – Yes 2 – No	
1.											
2.											
3.											
4.											
5.											
6.											
7.											
